# Neonatal Encephalopathy due to Glutaminase Deficiency in a Neonate

**DOI:** 10.1002/ccr3.9567

**Published:** 2024-11-17

**Authors:** Unnati Achanta, Shrinidhi Krishnan, Ashok Chandrasekaran, Robert Wilson S, Senthil Kumar Aiyappan, Subash Sundar

**Affiliations:** ^1^ Department of Paediatrics SRM Medical College Hospital and Research Centre Chengalpattu India; ^2^ Department of Neonatology SRM Medical College Hospital and Research Centre Chengalpattu India; ^3^ Department of Neurology SRM Medical College Hospital and Research Centre Chengalpattu India; ^4^ Department of Radiology SRM Medical College Hospital and Research Centre Chengalpattu India

**Keywords:** epileptic encephalopathy, glutaminase, neonatal seizures, neurometabolic disorder, newborn

## Abstract

Identifying neurometabolic disorders that lead to neonatal encephalopathy is difficult, and access to exome sequencing is a significant advantage in developing countries. We present a case of neonatal encephalopathy characterized by refractory seizures and significant apnea resulting from glutaminase deficiency, along with elevated levels of glutamine and glycine in the cerebrospinal fluid. Although the condition was fatal, it was possible to offer genetic counseling and recommendations for future pregnancies following exome sequencing.

## Graphical abstract

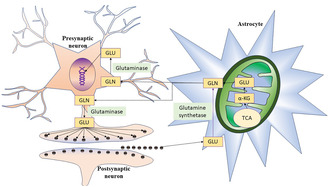

**Summary**

- Neurotransmitter glutamate is required for fetal brain development, synapsis formation, cellular metabolism, and regulation of respiration in postnatal period.
- Glutaminase enzyme deficiency in brain causes glutamate deficiency with high cerebrospinal fluid glutamine and glycine levels, leading to neonatal encephalopathy, refractory seizures, and severe apnea.
- Exome sequencing aids in the diagnosis of neurometabolic disorders presenting in newborn period.

## 1 Introduction

Developmental and epileptic encephalopathy 71 (DEE71) is a rare neurometabolic disorder caused by glutaminase enzyme deficiency (OMIM # 618328). Brain glutaminase catalyzes glutamine to glutamate conversion [1]. Glutamate is the primary excitatory neurotransmitter. Glutamate is essential for brain development, synapsis formation, neural functions, cellular metabolism, neuroplasticity, and respiration with inputs from peripheral chemoreceptors [2, 3, 4, 5, 6]. Glutamate is a precursor for gamma‐aminobutyric acid (GABA), an inhibitory neurotransmitter. Glutamate links carbohydrate and amino acid metabolism in the brain via the Krebs cycle [7]. Neurological impairment in children is caused by both gain of function and loss of function of glutaminase [8].

Developmental and epileptic encephalopathies (DEEs) are a heterogenous group of genetic conditions presenting with intractable seizures, impaired cognition, and developmental delay [9]. Availability of exome sequencing and advances in genotype–phenotype correlation methods have led to recognition of many DEEs [10]. DEE71 is caused by biallelic mutations of glutaminase gene with complete loss of glutaminase activity and presents in neonates with severe hypotonia, refractory seizures, apnea, gliosis, cerebral edema, and thinning of subcortical white matter of brain leading to early death [11]. Glutaminase loss of function caused by duplication or tandem repeats reported ataxia, optic atrophy, developmental delay, and progressive neurological deterioration in childhood [12, 13]. Glutaminase hyperactivity caused by heterozygous mutations was noticed to have profound developmental delay without dysmorphism, infantile cataract, and erythematic subcutaneous nodules [8]. Here, we report a neonate from India diagnosed with DEE71 caused by homozygous truncating mutation of glutaminase gene and a review of previous reported cases.

## 2 Case History and Examination

A term baby boy was delivered at 39 weeks by emergency section due to meconium‐stained amniotic fluid. He was second‐born to a consanguineously married couple with an uneventful pregnancy. He required prolonged positive pressure ventilation with intubation after birth and shifted to the neonatal intensive care unit for ventilation support. Mild acidosis with pH 7.16, base excess −12.5 was seen in cord blood gas. Apgar scores at 1, 5, 10, and 20 min were 4, 5, 6, and 6, respectively.

Family history revealed death of a sibling. His brother was delivered at a primary‐level hospital after an uncomplicated pregnancy and referred to a tertiary center for postresuscitation care. He remained ventilator dependent and succumbed to illness on the third month. MRI brain on fourth week showed ulegyria, gliosis in basal ganglia and midbrain. Genetic testing for spinal muscular atrophy by multiplex‐ligation probe amplification was negative. No further genetic testing was done for the elder sibling.

The anthropometry details of our patient revealed a birth weight of 2750 g at 11th centile and a head circumference of 34 cm at 54th centile, and the length 46 cm was at 3rd centile [14]. He had no dysmorphic features or neurocutaneous markers, ophthalmological examination was normal, and testes were descended. Neurological examination revealed generalized hypotonia, severe stupor with minimal response to pain, poor spontaneous activity, occasional eye opening, positive doll's eye response, normal pupillary light reflex, elicitable deep tendon reflexes, and cremasteric reflex. Chest X‐ray showed adequate lung expansion. He had minimal ventilation requirement with a mean airway pressure of 8 cm H_2_O and inspired oxygen of 0.25. He had normal cardiac function, and full enteral feeds were established by Day 5 through gavage feeding. He was extubated to nasal intermittent mandatary ventilation on Day 7 of life.

## 3 Management and Differential Diagnosis

In view of stupor and severe hypotonia, he was evaluated for neonatal encephalopathy [15]. The first line of investigations was done to rule out transient metabolic insults, sepsis, acidosis, and evaluation of hepatic, renal, and thyroid function. His blood glucose, electrolytes, calcium, magnesium, plasma ammonia, blood gas analysis, complete hemogram, liver function, renal function, and thyroid function tests were normal. Cerebrospinal fluid (CSF) glucose and protein levels were normal, urine ketones were negative, and bedside neurosonogram showed no features of intracranial bleed/stroke. Blood and CSF cultures were sterile, and cytomegalovirus was not detected in his urine by polymerase chain reaction.

**FIGURE 1:**
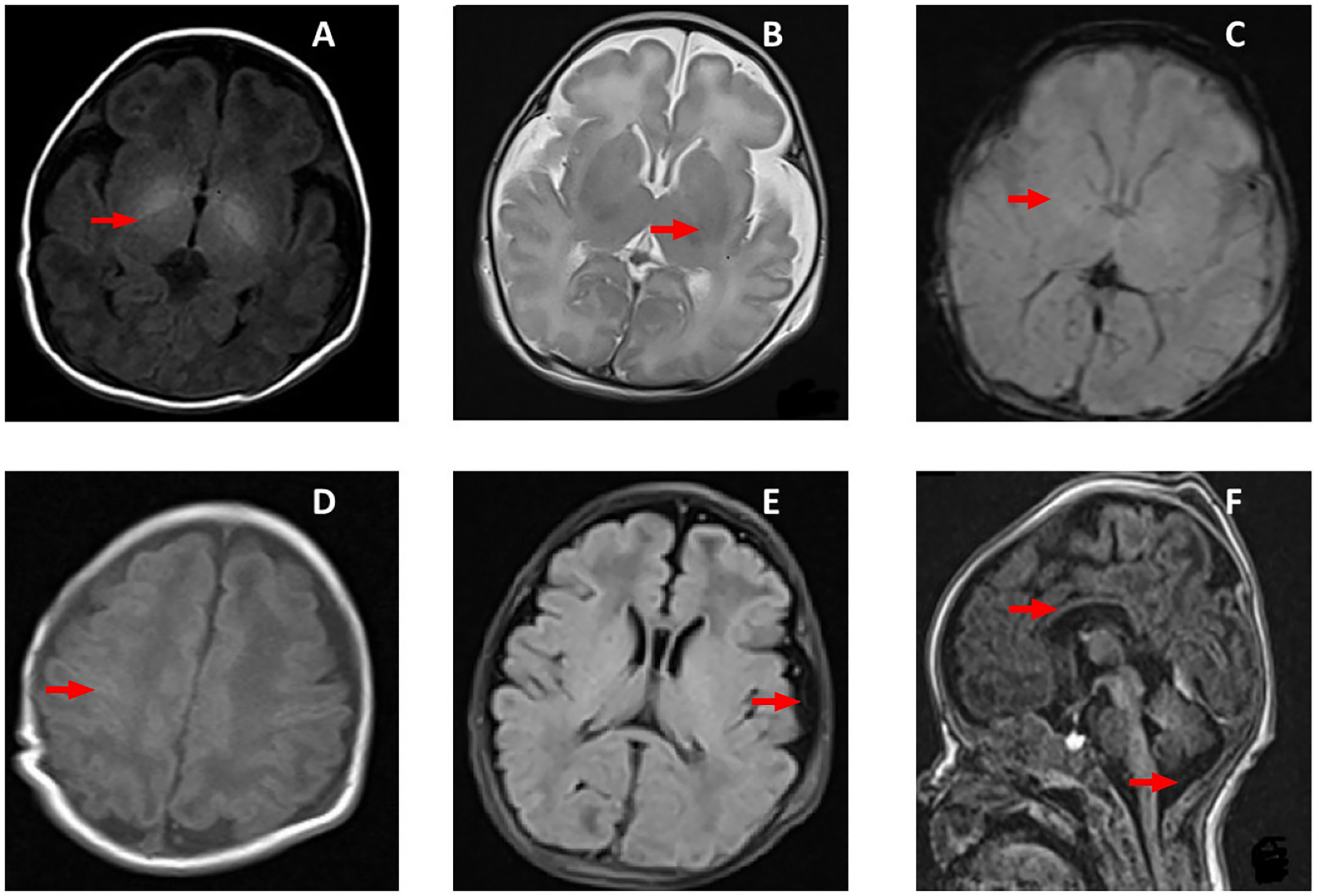
MRI images of the patient done on Day 5 of life. (A) Axial T1 image showing no myelination in posterior limb of internal capsule and high signals in basal ganglia. (B) Axial T2 image showing no myelination in posterior limb of internal capsule and normal signals in basal ganglia. (C) Axial gradient image showing no blooming and normal signals in basal ganglia. (B, C) Suggests absence of hemorrhage and vasogenic edema. (D) Image shows no myelination in perirolandic gyri. (E) Axial FLAIR image showing simplified cortical patterns and prominent sulcal spaces, and (F) Sagittal T1 images show thin corpus callosum and prominent cisterna magna.

Sibling history led to the suspicion of neuromuscular disorders. Levels of lactate dehydrogenase and creatine kinase–MB levels were within normal range at 278 (range: 160–450 Units/L) and 98 (range: 15–350 Units/L), respectively. MRI brain on Day 5 revealed hypoplastic corpus callosum, subcortical white matter loss in the fronto‐parietal area, a simplified gyral pattern, high signals in the basal ganglia on T1 images, a prominent cisterna magna, no diffusion restriction, and absence of myelination in the perirolandic gyri and posterior limb of the internal capsule (Figure 1). A neostigmine stimulation test was performed on 10th day in suspicion of congenital myasthenia or neonatal myasthenia syndrome variants showed no clinical improvement. His mother had no features of myotonia and myasthenia, and her acetylcholine receptor antibody profile was negative. On the second week of life, he remained hypotonic and required noninvasive ventilatory support and gavage feeding. Due to technical issues, nerve conduction studies, electromyography, and muscle biopsy were not performed.

**FIGURE 2:**
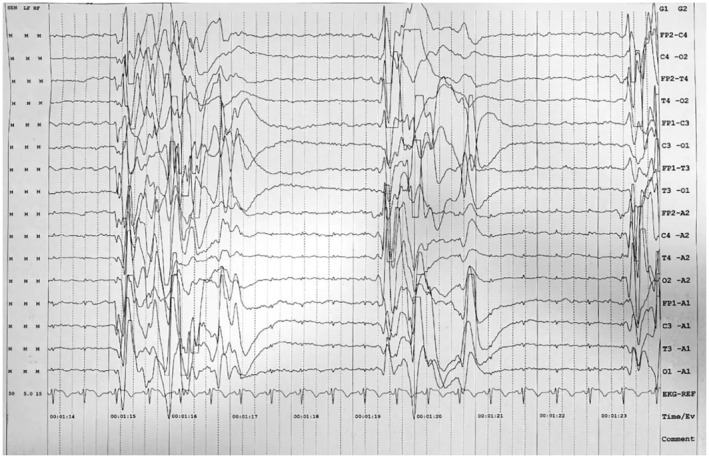
Electroencephalogram of the patient. Electroencephalogram of the patient in bipolar montage shows “burst suppression” pattern.

He developed refractory multifocal myoclonic seizures from Day 12, and electroencephalogram showed a “burst‐suppression” pattern (Figure 2). Workup for inborn error of metabolism in a referral lab showed normal plasma carnitine and acyl‐carnitine profiles. Plasma amino acid profile revealed high alanine levels 945 (range: 242–594 μmol/L) and high glycine levels 1584 (newborn range: 232–740 μmol/L). Additionally, glutamine and glutamate levels were abnormal. Low glutamine levels 135 (range: 450–850 μmol/L) and high glutamic acid levels 518 (range: 17–69 μmol/L) were reported (Table 1). However, given the known spontaneous conversion from glutamine to glutamate during the long transit time in sample transport, interpretation could not be done [16]. Qualitative urine gas chromatography–mass spectrometry showed elevated lactic acid and succinic acid levels indicating mitochondrial dysfunction. CSF amino acid profile revealed high glutamine 1507 (range: 231–638 μmol/L) and glycine 109 (range: 2–14 μmol/L) levels. CSF to plasma glycine ratio of 0.07 was in the range of attenuated nonketotic hyperglycemia [17, 18]. Oral sodium benzoate was added to lower his glycine levels. Anticonvulsant (phenobarbital, midazolam, levetiracetam, topiramate, phenytoin, pyridoxine, and folinic acid supplements) did not effectively manage seizures.

**TABLE 1:** Plasma and cerebrospinal fluid (CSF) amino acid levels by UHPLC.

Amino acid	Patient plasma levels (μmol/L)	Reference plasma levels [17, 18] (μmol/L)	Patient CSF levels (μmol/L)	Reference CSF levels [17, 18] (μmol/L)
Alanine	**945**	242–594	45	12.5–47.3
Arginine	41	1–81	6	5.9–30.6
Asparagine	31	28–65	10	< 23.6
Aspartic acid	43	34–94	43	6–18
Citrulline	17	19–52	2	< 5.6
Histidine	9	68–108	9	11–25
Cysteine	26	36–58	2	< 5
Glutamine	**135**	450–850	**1507**	231–638
Glutamic acid	**518**	17–69	5	< 15
Glycine	**1584**	232–740	**109**	2–14
Isoleucine	43	34–106	9	1–11
Leucine	133	86–206	15	3.4–25.9
Lysine	194	116–276	27	7.8–40.8
Methionine	100	13–60	43	0.4–9.4
Ornithine	86	47–195	6	1.6–12
Phenylalanine	84	34–86	14	6.9–25.1
Proline	149	58–324	154	< 8
Serine	168	92–196	53	18–73
Threonine	67	102–246	33	10.8–74.9
Tyrosine	54	35–107	28	5.4–23.7
Valine	96	155–343	15	10–38
Tryptophan	36	10–140	4	—

*Note:* Bold values indicate plasma amino acid profile showed elevated levels of alanine and glycine. Furthermore, reduced glutamine levels and elevated glutamic acid were observed. CSF amino acid profile showed high glutamine and high glycine 109 levels.

Abbreviation: UHPLC, ultra‐high‐performance liquid chromatography.

Based on the clinical features, history, and laboratory findings, we excluded sepsis, stroke, brain malformations, transient metabolic conditions, fatty acid oxidation defects, organic acidemias, urea cycle disorders, myotonic dystrophy, and transient myasthenia. Glycine encephalopathy, mitochondrial disorders, and neurometabolic disorders were included in differential diagnosis.

## 4 Genetic Tests

Karyotyping revealed a normal 46 XY pattern. Microarray by Affymetrix CytoScan 4.3.0.71 showed no copy number variation and homozygosity seen in 6.03% of the genome. Exome sequencing by the Illumina sequence platform on “Genome Reference Consortium38” revealed a homozygous mutation in the exon 3 of Glutaminase (GLS) gene (Chr2:g.190895625 C>T), which resulted in premature truncation of the protein at codon 169 (p.Arg169Ter). This variant was not reported in 1000Genomes, gnomAD, and other databases. In silico prediction by MutationTaster2 showed the variant is pathogenic [19]. This mutation was confirmed by Sanger sequencing, and both parents were carriers of the mutation.

In this case, DEE 71 was caused by a homozygous truncating mutation of glutaminase gene, which was identified in whole‐exome sequencing and confirmed by Sanger sequencing. He succumbed to status epilepticus on Day 48.

## 5 Discussion

Determining the etiology of neonatal encephalopathy aided in optimal management of this case [20]. Our patient required resuscitation at birth, but cord blood gas was not suggestive of severe asphyxia. After initial workup, we had excluded infection, perinatal asphyxia, feto‐maternal hemorrhage, intracranial bleed, ischemic stroke, major malformation, hyperammonemia, organic acidemia, common inborn error of metabolism with intoxication, and transient metabolic conditions. We could not conduct further neurophysiological studies due to nonavailability of appropriate electrodes, and the transfer of muscle biopsy specimens to nearest referral center was not practical [21, 22]. Intractable seizures, hypotonia, consanguinity, sibling death, and normal neurological examination of his mother directed toward genetic, neurometabolic, or mitochondrial causes. Exome sequencing aids in early diagnosis of monogenic causes of neonatal encephalopathy, and DEE 71 was diagnosed in our patient [9, 23].

**FIGURE 3:**
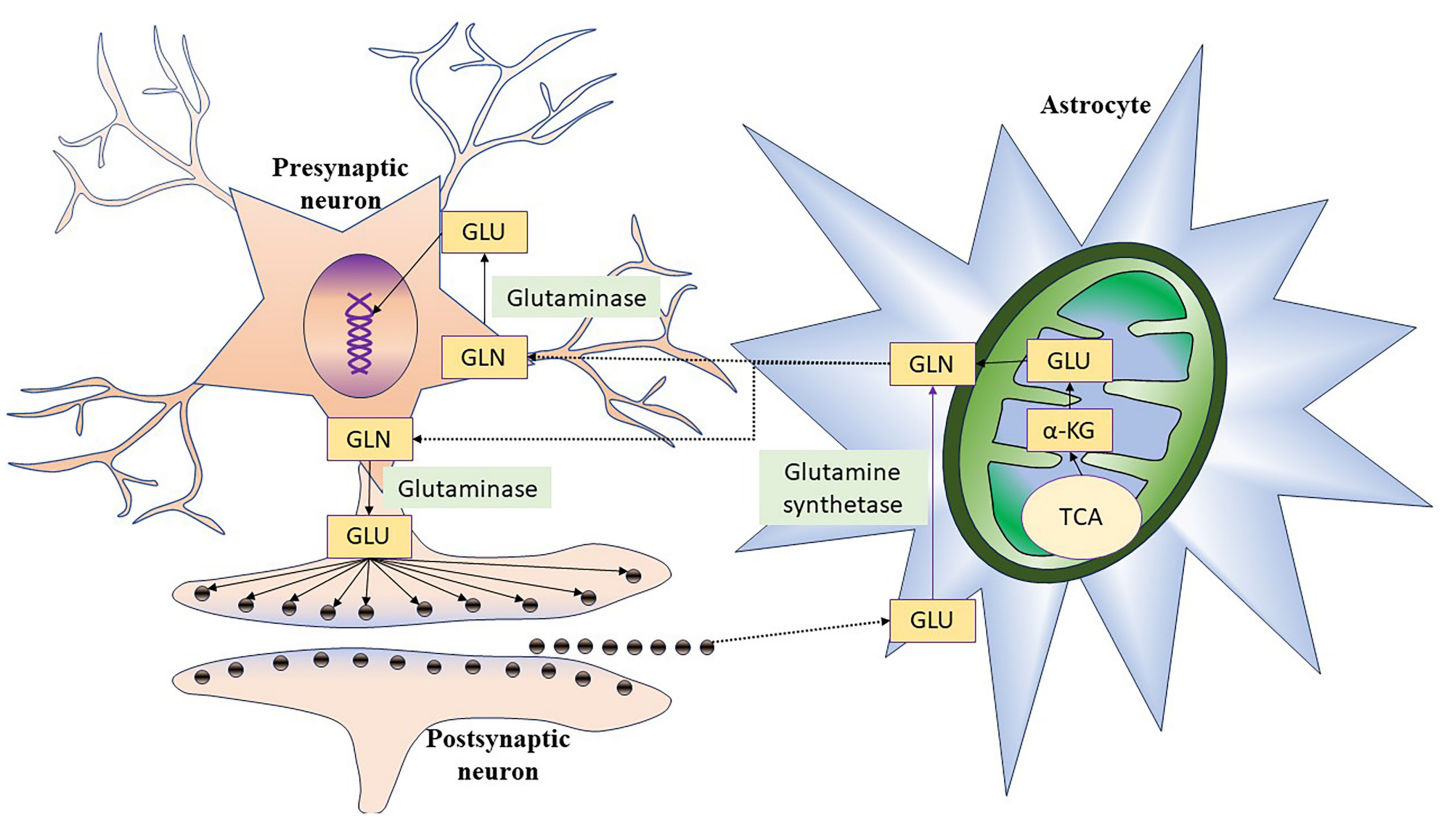
Glutamine and glutamate shuttle between astrocytes and neurons. Astrocytes take glutamate (GLU) from neuronal synapsis or produced in mitochondria by tricarboxylic acid cycle (TCA). GLU is converted to glutamine (GLN) by glutamine synthetase in astrocytes. GLN is transported to neurons for conversion to GLU by glutaminase enzyme. GLU helps in nucleic acid production and neurotransmission.

Glutamate metabolism in the brain is complex and localized. Glutamine–glutamate shuttle is maintained by the interplay of neurons and astrocytes [1]. Astrocytes uptake glutamate from synaptic vesicle or synthesize glutamate in mitochondria through the Krebs cycle from α‐ketoglutarate by the enzyme aspartate aminotransferase. Glutamine is produced in astrocytes by the action of glutamine synthetase on glutamate. Glutamine from astrocytes is transferred to neurons, and neurons synthesize glutamate by the action of glutaminase enzyme (Figure 3). Neurons use glutamate in signal transduction [7]. In synapses, glutaminergic neurons release glutamate, while in GABAergic neurons, glutamate decarboxylase converts glutamate to GABA. Thus, glutamate is critical for both excitatory and inhibitory neurotransmission in brain [7, 24].

GLS gene is highly expressed in brain, and glutaminase deficiency shows the importance of delicate balance required to maintain glutamine–glutamate shuttle [7]. Glutaminase deficiency causes high cellular glutamine levels, which leads to osmotic dysfunction, edema, and necrotic cell death [25]. Glutamate is essential for cellular energy metabolism, signal transmission, and myelin formation [3]. This could explain the lack of myelination, hypoplastic corpus callosum, loss of subcortical white matter, simplified gyral pattern, and prominent cisterna magna in our case. High signals in basal ganglia on T1 images could have been due to edema, and normal signals on T2 and gradient images confirmed the absence of hemorrhage.

The previous case reports of biallelic GLS gene mutation in 4 infants causing DEE71 showed similar findings with lethal apnea, hypotonia, and intractable seizures (Table 2). In these reports, dried blood spot analyzed by modified mass spectrometry showed high glutamine and normal glutamate levels. But, plasma amino acid analysis of our patient by high‐performance liquid chromatography revealed low glutamine and high glutamic acid levels. In developing countries, there is a lack of immediate access to advanced biochemical testing labs, resulting in the need to transport blood samples to the nearest referral lab [26]. Our patient's samples were sent in cold storage via fast courier, but samples were analyzed only after 3 days of collection. This could have resulted in heat exposure of the sample and misinterpretation of the plasma amino acid assay of our patient. Sample exposure to 22°C for 2 h could cause a 5‐fold rise in glutamic acid levels caused by cellular protein degradation, anaerobic deamidation of glutamine to glutamate, and a decrease in glutamine level [16]. Additional alterations in plasma amino acid due to heat exposure of sample include a decrease in cysteine and aspartate levels, as well as an increase glycine, alanine, and asparagine levels. High alanine levels are also caused by disorders of mitochondria. CSF amino acid profile of our patient showed high glutamine levels and low glutamic acid levels consistent with previous case reports. This might be attributed to lower cellular protein content in CSF, and transport had little impact on interpretation. To our knowledge, CSF amino acids were not described in previous case reports. Anticonvulsant therapy and mitochondrial dysfunction could increase CSF glycine levels [27]. Magnetic resonance spectroscopy was not done in our patient.

**TABLE 2:** Comparison of clinical and genetic details of previously reported cases and our patient.

Characteristics	Previous reported family 1 [11]		Previous reported family 2 [11]		Our patient's family 3	
	Sibling 1	Sibling 2	Sibling 1	Sibling 2	Sibling 1	Sibling 2
Consanguinity	Yes		No		Yes	
Birth weight^a^ grams, Centile	Unknown	3040 43rd	2990 8th	3000 11th	3010 28th	2750 11th
Length^a^ cm, centile	Unknown	49 25th	47 1st	47 1st	48 22nd	46 3rd
Head circumference^a^ cm, centile	Unknown	36.5 90th	Unknown	32 1st	34 54th	34 54th
Apgar scores	Unknown					
1 min		6	2	4	2	4
5 min		5	7	5	4	5
10 min		7	7	7	6	6
Seizure onset/type	Similar to affected sibling	10 min of life/focal seizures	From Day 2/Focal seizures on Day 2, then asymmetric tonic, eyelid clonus, irregular eye movement, myoclonic jerks	Myoclonic jerks from Day 2	Day 1/Multifocal clonic seizures, then myoclonic jerks	Day 10/Myoclonic jerks
Seizure response to therapy	Refractory	Refractory	Refractory	Refractory	Refractory	Refractory
Seizure medications	Unknown	Lorazepam, levetiracetam, phenobarbitone, phenytoin, steroid, valproate, pyridoxine, ketamine, pyridoxine6 phosphate, vigabatrin	Phenobarbitone, phenytoin, pyridoxine, midazolam, topiramate	Levetiracetam, phenobarbitone, vigabatrin, pyridoxine, pyridoxine6 phosphate	Phenobarbitone, phenytoin, levetiracetam, steroid, pyridoxine, biotin, topiramate	Levetiracetam, phenobarbitone, phenytoin, midazolam, pyridoxine, folinic acid, topiramate
Electroencephalogram	Unknown	Burst‐suppression	Burst‐suppression, variable focal onset seizures with α/β activity	Burst‐suppression, generalized rhythmical seizures	Predominantly burst‐suppression	Burst‐suppression
Muscular tone	Unknown	Hypotonia	Hypotonia	Hypotonia	Hypotonia	Hypotonia
Respiratory function	Respiratory insufficiency	Poor respiratory efforts need ventilator support	Poor respiratory efforts, Cheyne‐Stokes breathing	Respiratory insufficiency, apnea need ventilator support	Ventilator dependent, apneic	Poor respiratory efforts need ventilator support
MRI brain time, days	Unknown	0 and 30	3	3	28	5
MRI brain findings	Unknown	Initial simplified gyral patten, anterior to posterior gradient and subcortical brain involved. Follow‐up scans show gliosis, loss of volume in basal ganglia, corpus callosum, thalamus, brainstem and vermis with vasogenic edema	Simplified gyral pattern of the frontal lobes with white matter involvement	Severe demyelination with calcium spots and recess of subcortical white matter	Ulegyria with shrunken cortex and gliosis of basal ganglia, mid brain regions	Absence of myelination in posterior limb of internal capsule, perirolandic gyri, prominent cisterna magna, simplified gyral pattern, hypoplastic corpus callosum
Outcome	Mortality	Mortality	Mortality	Mortality	Mortality	Mortality
Dysmorphic features	Unknown	None	None	None	None	None
Genetic position/Genome Variation	Not tested	Chr2:191765378/c.695dup	Chr2:191766752 & Ch2:191746051/c.815G>A & c.241 C>T	Chr2:191766752 & Ch2:191746051/c.815G>A & c.241 C>T	Not tested	Chr2:190895625/c.505 C>T
Protein alteration	Not tested	p.(Asp232Glufs*2)	p.(Arg272Lys)/p.(Gln81*)	p.(Arg272Lys)/p.(Gln81*)	Not tested	p.(Arg169Ter)
Zygosity	Not tested	Homozygous	Compound heterozygous	Compound heterozygous	Not tested	Homozygous

Abbreviation: MRI, magnetic resonance imaging.

^a^ Anthropometry details at birth.

Milder genetic changes in GLS gene with loss of function caused by tandem repeat changes of the noncoding area led to early‐onset developmental delay, ataxia, cerebellar atrophy, and gradual neurological deterioration in early childhood [12, 28]. Glutaminase deficiency caused by duplication in an exon of the GLS gene led to spastic ataxia, optic atrophy, and degenerative disorder with loss of motor and language skills in a young child [13]. Similarly, gain of function of glutaminase caused by heterozygous missense variants in GLS gene is reported. Glutaminase hyperactivity has led to progressive developmental delay in children, and cataract, no dysmorphic features, generalized tonic–clonic seizures, and magnetic resonance spectroscopy have shown higher glutamate/glutamine ratio [8, 29]. These case reports illustrate significance of glutamine–glutamate shuttle in regulating normal glutamate levels of brain for normal neurological development in children.

Advances in genetic testing, ease of availability, and decrease in cost of exome sequencing technique have helped in establishing the diagnosis of sick children in developing countries for suspected monogenic disorders [30, 31]. Genomic methods are powerful tools to analyze early onset neurometabolic disorders. The availability of genome sequencing has enabled us to identify many DEEs [23]. Rumping et al. first reported DEE 71 in two families with early‐onset neonatal refractory seizures, respiratory failure, structural brain changes, cerebral edema, high glutamine levels in blood, death in early infancy, and exome sequencing revealed biallelic GLS gene mutation [11]. DEE 71 caused by GLS deficiency was recognized as an entity from 2019 (OMIM # 618328). The comparison of genetic mutations in our patient and previously reported cases is shown (Table 2). His elder brother possibly had glutaminase deficiency and “double trouble” pathology with added hypoxic insult could explain the MRI [18], but samples were not available for confirmation.

## 6 Conclusion

In our patient, access to genomic sequencing assisted in diagnosing DEE71 and providing genetic counseling to the parents, despite challenges such as lack of neurophysiological study facilities and nearby labs for advanced testing of inborn error of metabolism. Cases with this disorder would be missed if genetic panels are not included, so it is recommended to test the first affected family members. To our knowledge, this is the first case of DEE71 reported from India, caused by a homozygous nonsense mutation in GLS gene at Chr2:g.190895625 C>T resulting in premature termination of protein at codon 169 (p.Arg169Ter). This loss of function of GLS led to abnormalities in MRI brain, burst suppression pattern in electroencephalogram, elevated glutamine levels in CSF, refractory seizures, hypotonia, severe apnea requiring respiratory support, status epilepticus, and invariably a fatal outcome.

## Author Contributions

**Unnati Achanta:** conceptualization, methodology, project administration, writing – original draft. **Shrinidhi Krishnan:** formal analysis, investigation, resources, writing – original draft. **Ashok Chandrasekaran:** conceptualization, data curation, formal analysis, investigation, methodology, software, supervision, validation, visualization, writing – review and editing. **Robert Wilson S:** investigation, supervision, visualization, writing – review and editing. **Senthil Kumar Aiyappan:** investigation, visualization, writing – review and editing. **Subash Sundar:** project administration, supervision, visualization, writing – review and editing.

## Consent

Written consent was obtained from both parents of the case in appropriate consent forms for publication of clinical information and radiological/photographical material without patient identifier details.

## Conflicts of Interest

The authors declare no conflicts of interest.

## Data Availability

The data used in this case report shall be shared upon by reasonable request to the corresponding author.
